# SAFIR-I: first NEMA NU 4-2008-based performance characterization

**DOI:** 10.1186/s40658-023-00603-1

**Published:** 2023-12-12

**Authors:** Pascal Bebié, Werner Lustermann, Jan Debus, Christian Ritzer, Günther Dissertori, Bruno Weber

**Affiliations:** 1 Institute for Particle Physics and Astrophysics, ETH Zurich, Otto-Stern-Weg 5, 8093 Zurich, ZH Switzerland; 2 Institute of Pharmacology and Toxicology, University of Zurich, Winterthurerstrasse 190, 8057 Zurich, ZH Switzerland; 3 Neuroscience Center Zurich, University and ETH Zurich, Winterthurerstrasse 190, 8057 Zurich, ZH Switzerland

**Keywords:** Positron Emission Tomography, PET/MRI, Preclinical imaging, SAFIR, NEMA NU 4-2008, Performance characterization

## Abstract

**Background:**

Small Animal Fast Insert for MRI detector I (SAFIR-I) is a novel Positron Emission Tomography insert for a $7\,\hbox {T}$ Bruker BioSpec 70/30 Ultra Shield Refrigerated Magnetic Resonance Imaging (MRI) system. It facilitates truly simultaneous quantitative imaging in mice and rats at injected activities as high as $500\,\hbox {MBq}$. Exploitation of the resulting high count rates enables quick image formation at few seconds per frame. In this investigation, key performance parameters of SAFIR-I have been determined according to the evaluations outlined in the National Electrical Manufacturers Association (NEMA) Standards Publication NU 4-2008 (NEMA-NU4) protocol.

**Results:**

Using an energy window of 391 to $601\,\hbox {keV}$ and a Coincidence Timing Window of $500\,\hbox {ps}$, the following performance was observed: The average spatial resolution at $5\,\hbox {mm}$ radial offset (Full Width at Half Maximum) is $2.54\,\hbox {mm}$ when using Filtered Backprojection, 3D Reprojection reconstruction. For the mouse- and rat-like phantoms, the maximal Noise-Equivalent Count Rates (NECRs) are $1368\,\hbox {kcps}$ at the highest tested average effective concentration of $14.7\,\hbox {MBq}\,\hbox {cc}^{-1}$, and $713\,\hbox {kcps}$ at the highest tested average effective concentration of $1.72\,\hbox {MBq}\,\hbox {cc}^{-1}$, respectively. The NECR peak is not yet reached for either of these cases. The peak sensitivity is $1.46\,\%$. The Image Quality phantom uniformity standard deviation is $4.8\,\%$. The Recovery Coefficient for the $5\,\hbox {mm}$ rod is $(1.08 \pm 0.10)$. The Spill-Over Ratios are $(0.22 \pm 0.03)$ and $(0.22 \pm 0.02)$, for the water- and air-filled cylinder, respectively. An accuracy of $4.3\,\%$ was achieved for the quantitative calibration of reconstructed voxel values.

**Conclusions:**

The measured performance parameters indicate that the various design goals have been achieved. SAFIR-I offers excellent performance, especially at the high activities it was designed for. This facilitates planned experiments with fast tracer kinetics in small animals. Ways to potentially improve performance can still be explored. Simultaneously, further performance gains can be expected for a forthcoming insert featuring 2.7 times longer axial coverage named Small Animal Fast Insert for MRI detector II (SAFIR-II).

## Background

The Small Animal Fast Insert for MRI (SAFIR) collaboration is developing novel Positron Emission Tomography (PET) inserts for a $7\,\hbox {T}$ Bruker BioSpec 70/30 Ultra Shield Refrigerated (USR) Magnetic Resonance Imaging (MRI) system [1, 2]. The inserts are specified to enable quantitative, truly simultaneous PET/MRI research in mice and rats, with a spatial resolution of around $2\,\hbox {mm}$ in the center of the Field Of View (FOV), at injected activities extending up to $500\,\hbox {MBq}$. Exploitation of the associated high count rates, specifically the corresponding low noise, enables an exceptional image frame acquisition speed in the order of one diagnostically relevant frame every few seconds [1, 3, cf.]. This high frame rate is required for capturing fast tracer kinetics, which was not possible with existing small-animal PET systems [1, cf.].

A first insert, Small Animal Fast Insert for MRI detector I (SAFIR-I), has successfully been constructed and commissioned [1]. In this study, fundamental performance parameters of SAFIR-I have been determined according to the NEMA Standards Publication NU 4-2008 (NEMA-NU4) protocol.

### The SAFIR-I detector design

The complete design and functionality of the detector have previously been described in [1]. Additional material on the data acquisition software and the analysis software can be found in [3–5]. The following paragraphs summarize the key information from those sources.

**Fig. 1:**
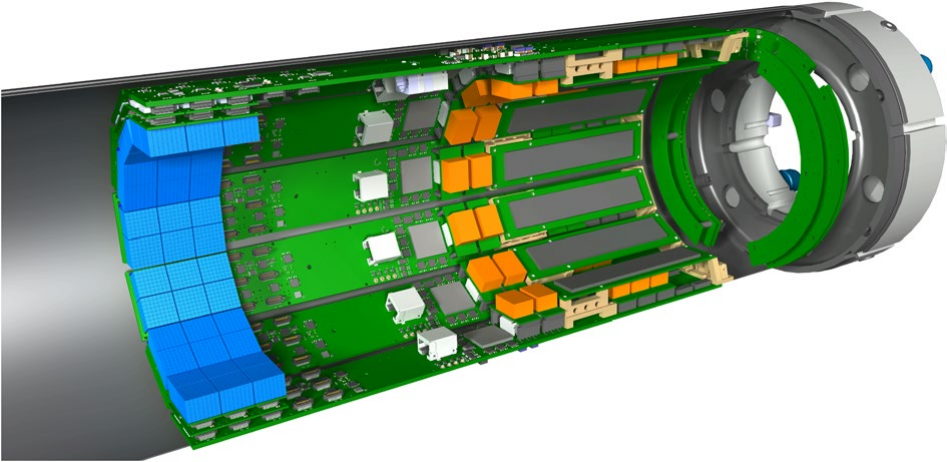
Cutaway view of SAFIR-I. Seven detector sections are displayed. The detector head is shown on the left hand side of the image; the three pairs of crystal matrices per section are depicted in light blue. Rendering by R. Becker for the SAFIR collaboration

**Fig. 2:**
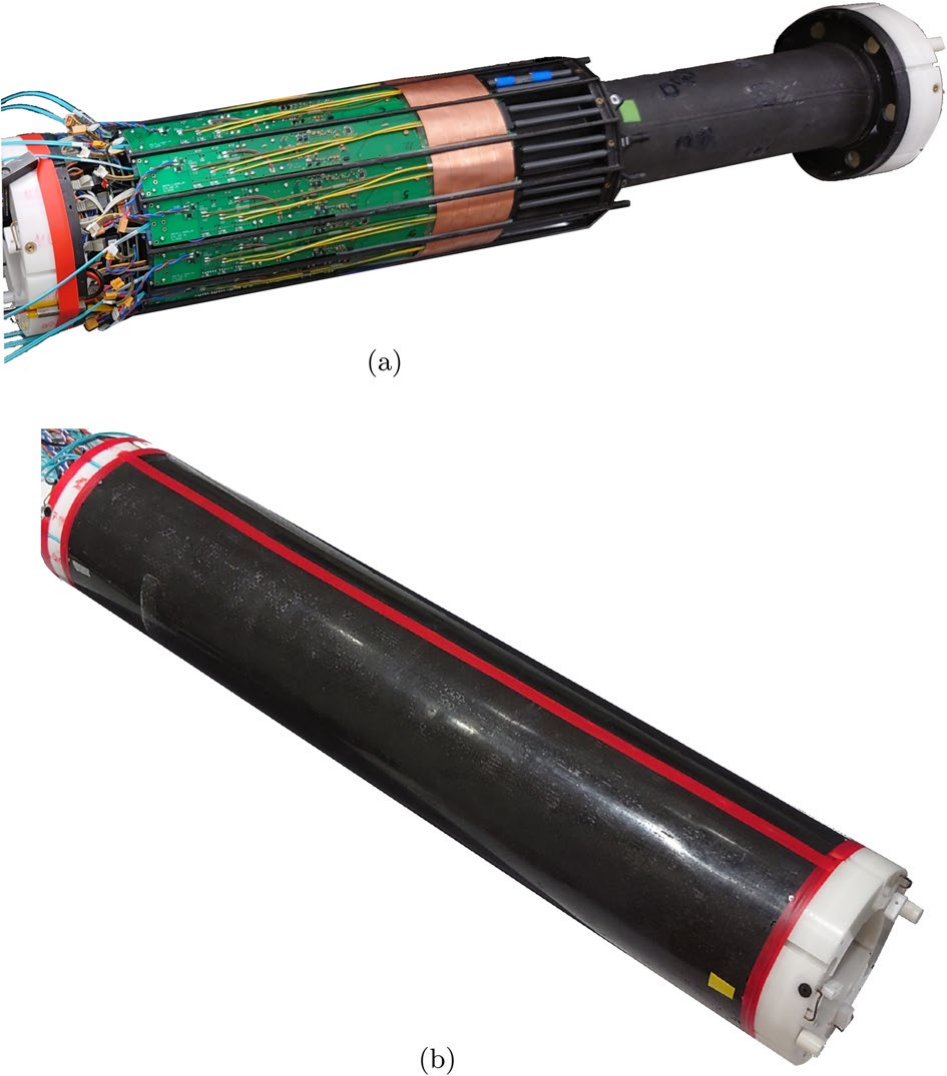
Photographs of the open and closed SAFIR-I detector. Photography by P. Bebié

SAFIR-I, shown in Figs. 1 and 2, has a dodecagonal shape with an outer diameter of $198\,\hbox {mm}$, an inner diameter of $114\,\hbox {mm}$ and an axial FOV of $54.2\,\hbox {mm}$. The detector head comprises 24 rings of Lutetium Yttrium OxyorthoSilicate (LYSO) crystals (Sichuan Tianle Photonics) with dimension $2.12\,\hbox {mm}\,\times \,2.12\,\hbox {mm}\,\times 13\,\hbox {mm}$, which are separated by Enhanced Specular Reflector (ESR) foils (3 M), assembled into pairs of matrices of $8 \times 8$ and $7 \times 8$ crystals (due to spatial constraints) at a pitch of $2.2\,\hbox {mm}$. Three such pairs are mounted in series in axial direction at a pitch of $18.1\,\hbox {mm}$, effectively forming three rings of crystal blocks, and hence summing up to 4320 crystals in the detector head in total. Due to the weakly radioactive (half life of $(4.05 \pm 0.09) \times 10^{10}\,\hbox {year}$ [6]) $^{176}$Lu contained in the crystal material, contributions from the intrinsic radioactivity of the detector have to be considered explicitly below.

The crystal matrices are read out by Silicon Photomultiplier (SiPM) arrays (Hamamatsu S13361-2050AE-08 SPL0) matching the crystal matrices’ pitch. The signals are digitized by Application Specific Integrated Circuits (ASICs) (Position-Energy-Timing Application Specific Integrated Circuit, version 6, Single Ended (PETA6SE) [7, 8], 144 in total), subsequently processed by a Field-Programmable Gate Array (FPGA) (Xilinx Kintex-7 XC7K70T) in every detector section and then passed to a Data Acquisition (DAQ) computer through $1\,\hbox {GBit}$ Ethernet connections.

The data processing including all calibration steps is described in [1, 4]. Coincidences are saved in listmode files subsequently used for reconstruction and data analysis according to NEMA-NU4.

## Methods

All measurements reported in this manuscript have been taken with SAFIR-I. The PET insert was permanently installed inside the constant magnetic field of the MRI system during all tests. No Magnetic Resonance (MR) coils were used concurrently and no simultaneous MR acquisitions took place to preclude excessive experimental complexity. Full MR-compatibility of SAFIR-I has previously been shown [1]. A well counter dose calibrator (Medisystem^1^ Medi 405) was used to determine the activities of the employed sources.

The data collection, processing, and analysis followed the NEMA-NU4 protocol (for convenience simply denoted “the protocol” henceforth) [9], with minimal adaptations detailed in subsections – below. For the coincidence search, a CTW of $500\,\hbox {ps}$ and an energy window of 391 to $601\,\hbox {keV}$ [1, 4] were applied in all cases. All reconstructions were performed using methods implemented in Software for Tomographic Image Reconstruction (STIR) [10], with a voxel size of $0.55\,\hbox {mm}\times 0.55\,\hbox {mm}\times 1.1\,\hbox {mm}$.

The performance reports for each test were again based on the protocol’s instruction. Finally, the achieved performance results were compared to the performance parameters reported for a set of reference scanners featuring similarly sized crystals, including the SAFIR prototype system, as tabulated in [3].

### Spatial resolution

**Fig. 3:**
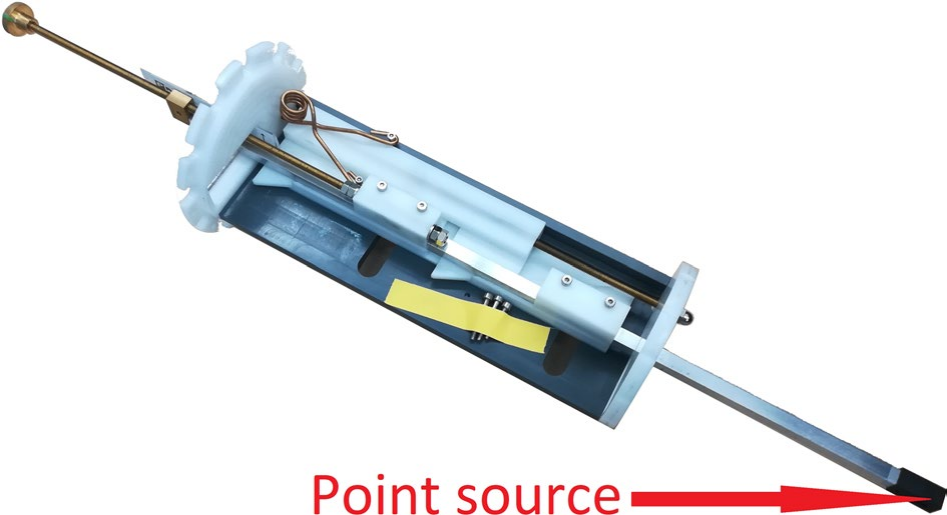
Point source holder for SAFIR-I. The radius of the white alignment plates corresponds to the inner radius of the detector; the 3D-printed tip (black) accepts $1-\hbox {cm}^{3}$ cubic sources ($^{22}$Na) for detector calibration and NEMA-NU4 measurements. Design by R. Becker for the SAFIR collaboration

A $^{22}$Na point source embedded in a $1-\hbox {cm}^{3}$ acrylic cube (Eckert & Ziegler Isotope Products, High-Resolution Marker “NEMA,” MMS09-022, source diameter $0.25\,\hbox {mm}$) was used. Its activity was $316.3\,\hbox {kBq}$, a level at which dead time losses are negligible for SAFIR-I. As demanded by the protocol, the axial source positions were $0\,\hbox {mm}$ (axial center) and $13.5\,\hbox {mm}$ (one quarter of the axial FOV). Supplementary to the mandatory offset evaluations, measurements at $0\,\hbox {mm}$ radial offset were taken. At each source location, $6 \times 10^{5}\,\hbox {coincidences}$ were collected to satisfy the requirement of acquiring at least $10^5$ coincidences. Instead of the animal bed on a single-direction rail, a point source holder facilitating point source positioning in two directions was used, as shown in Fig. 3.

The data analysis was performed using STIR’s FBP3DRP reconstruction algorithm without any filtering or smoothing of the data (as stipulated). As a back-projection filter the STIR-default Colsher filter ($\alpha = 1$, cut-off 0.5 cycles in both axial and planar directions) was used. A known downside of this algorithm is its requirement for cylindrical projection data; for a detector with block geometry like SAFIR-I, direct interpolation of the data to fit the cylindrical geometry leads to image degradation with severe streak artifacts [3, 5, 11, 12]. In order to partially ameliorate the degradation, the raw data from SAFIR-I were first sorted into projection data using its true, generic block geometry and then rebinned into a cylindrical projection [5, 11, cf.], prior to reconstruction.

### Scatter fraction, count losses, and random coincidence measurements

**Fig. 4:**
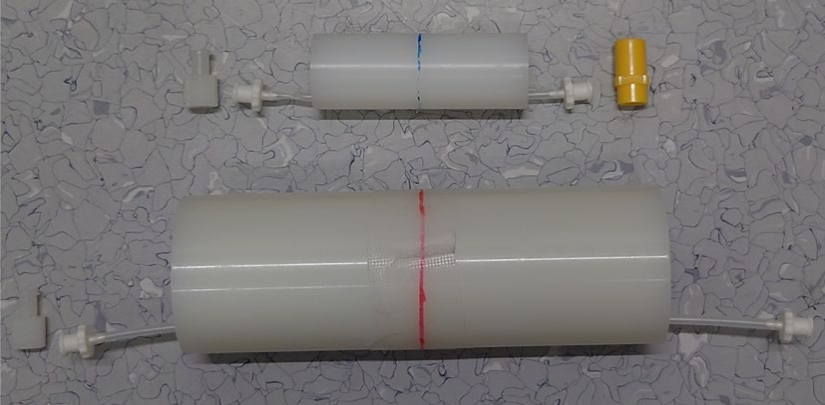
The NECR phantoms used. Top: Mouse-like. Bottom: Rat-like. Standard Luer lock connectors and plugs on both ends enabled to fill and seal the active volume

As SAFIR-I can fit mice and rats, scatter phantoms for those two use-cases were obtained (QRM Micro-PET Scatter Phantom mouse size / rat size, see Fig. 4).

For measurements of the background due to intrinsic radioactivity of SAFIR-I’s LYSO crystals, $42\,\hbox {h}$ and $48\,\hbox {h}$ of continuous data could be acquired, for the mouse-like and rat-like phantom, respectively.

For the acquisition of count rate data, both phantoms were filled with a solution of $^{18}$F in water. In the case of the mouse-like phantom the liquid volume was $0.225\,\hbox {mL}$; in the case of the rat-like phantom it was $0.450\,\hbox {mL}$.

In order to get a good resolution on any count rate peak potentially presenting at high activities, 16 measurements were taken at shorter $600\,\hbox {s}$ intervals, before the interval time was extended to $3200\,\hbox {s}$ for the remaining 18 measurements. The acquisition time for the first measurement was $1\,\hbox {s}$ and for subsequent acquisitions it was exponentially increased to the nearest full second to compensate for the decay. For these experiments, the start and end activities for the mouse-like phantom were $506.1\,\hbox {MBq}$ and $580.2\,\hbox {kBq}$; for the rat-like phantom they were $506.1\,\hbox {MBq}$ and $564.3\,\hbox {kBq}$, respectively.

The scatter fraction was determined for the last data point in each case. The protocol’s instruction was followed in all other points.

### Sensitivity

The same $^{22}$Na point source as in subsection was used, at an activity of $313.6\,\hbox {kBq}$. Per measurement point, $8 \times 10^{4}$ coincidences were acquired in order to obtain a smoother axial sensitivity curve [3, cf.].

Furthermore, the point source holder (see Fig. 3) only allowed for a step width of an estimated $(1.0 \pm 0.1)\,\hbox {mm}$, as opposed to the step size of one slice thickness (i.e., $1.1\,\hbox {mm}$) which the protocol asked for. Hence, the axial FOV was covered in 55 instead of 49 steps. To account for the extra steps when going in either direction from the axial center in the sum calculation of the total system sensitivity, all sensitivities but the central value were weighted with a factor of 48/54. Furthermore, considering the uncertainty associated with the mechanical setting of the source position, the positions were determined from the data.

In all other aspects, the steps outlined by the protocol were followed.

### Image quality, accuracy of attenuation, and scatter corrections

**Fig. 5:**
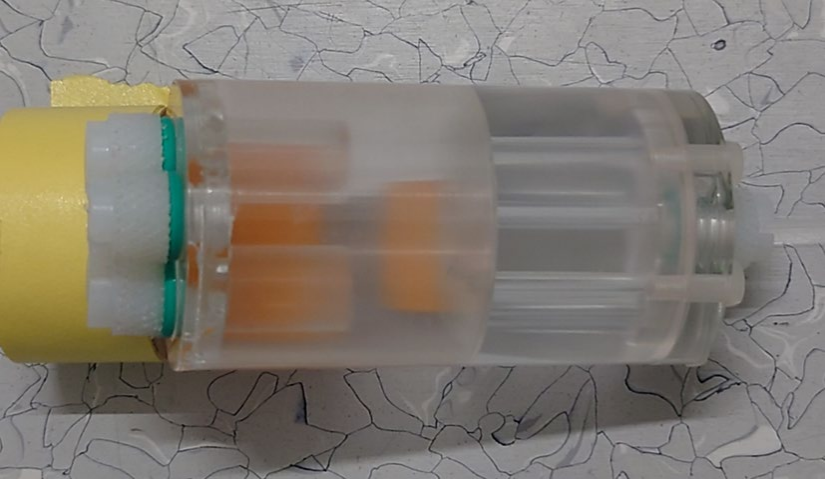
The IQ phantom used. Markers for rapid positioning and alignment were added on orange tape. Yellow tape (left) was used to fix the phantom in place on the animal bed

An IQ phantom was obtained (QRM Micro-PET IQ Phantom, see Fig. 5) and its active volume filled with a solution of $^{18}$F in water. Due to a temporary issue with the well counter, the start activity was $3.35\,\hbox {MBq}$, i.e., marginally below the protocol-requested $3.52\,\hbox {MBq}$. The acquisition time was $20\,\hbox {min}$. The image data were random, attenuation, scatter and normalization corrected [5, cf.]; the Singles-Prompts (SP) method [13] was used for the estimation of randoms and the Single Scatter Simulation (SSS) method [14, 15] was used to estimate scatter (see [5] for details). The attenuation maps were generated manually using STIR’s *generate_image* utility in conjunction with reconstructed measurement frames for reference, and including the known attenuation properties of the phantom materials. This approach is similar to the segmentation method [16]. A quantitative calibration of voxel values was achieved by imaging a cylindrical phantom of comparable dimensions to the IQ phantom but without any internal structures, hence larger active volume. Accordingly, the start activity was increased to $6.66\,\hbox {MBq}$ for the calibration phantom measurement in order to achieve a similar average activity concentration for an identical acquisition time of $20\,\hbox {min}$.

The images were reconstructed using a Maximum-Likelihood Expectation- Maximization (MLEM) algorithm with 30 iterations and a Gaussian inter-update filter with FWHM of $1.1\,\hbox {mm} \times 1.1\,\hbox {mm} \times 2.2\,\hbox {mm}$, i.e., twice the edge lengths of the reconstructed voxels, was applied [3, cf.]. The voxel value calibration factor CF was determined by inspecting a centered cylindrical Volume Of Interest (VOI) inside the calibration phantom with a radius of $75\,\%$ the phantom’s inner radius to prevent edge effects. There, the mean reconstructed number of counts was compared with the expected number of decays leading to the emission of $511\,\hbox {keV}$ photons based on the known activity, i.e.,

1 $$\begin{aligned} \mathrm{{CF}} = \frac{\text {expected no. of decays}}{\text {reconstructed counts}}. \end{aligned}$$

An $^{18}$F-to-$\beta ^{+}$ branching ratio of $96.86\,\%$ [17] was used for the calculation. This factor was subsequently applied to the IQ phantom data, leading to a calibrated number of reconstructed counts (denoted “calibrated counts” hereafter). To test the calibration, cylindrical VOI was drawn inside the central uniform region of the IQ phantom (again centered on the phantom’s axis and with a radius of $75\,\%$ the phantom’s inner radius). The accuracy *A* (in percent) of the calibration could then be estimated by comparing the mean of the calibrated counts in the VOI with the expected number of decays in the same volume, according to [3, 5, cf.]

2 $$\begin{aligned} A = \frac{\text {expected no. of decays}~-~\text {calibrated counts}}{\text {expected no. of decays}}~\times ~100\,\%. \end{aligned}$$

The procedures described in the protocol were followed in all remaining points, including the definitions of Regions Of Interest (ROIs)/VOIs, and the determinations of Spill-Over Ratio (SOR) and Recovery Coefficient (RC) values.

## Results

### Spatial resolution results

**Fig. 6:**
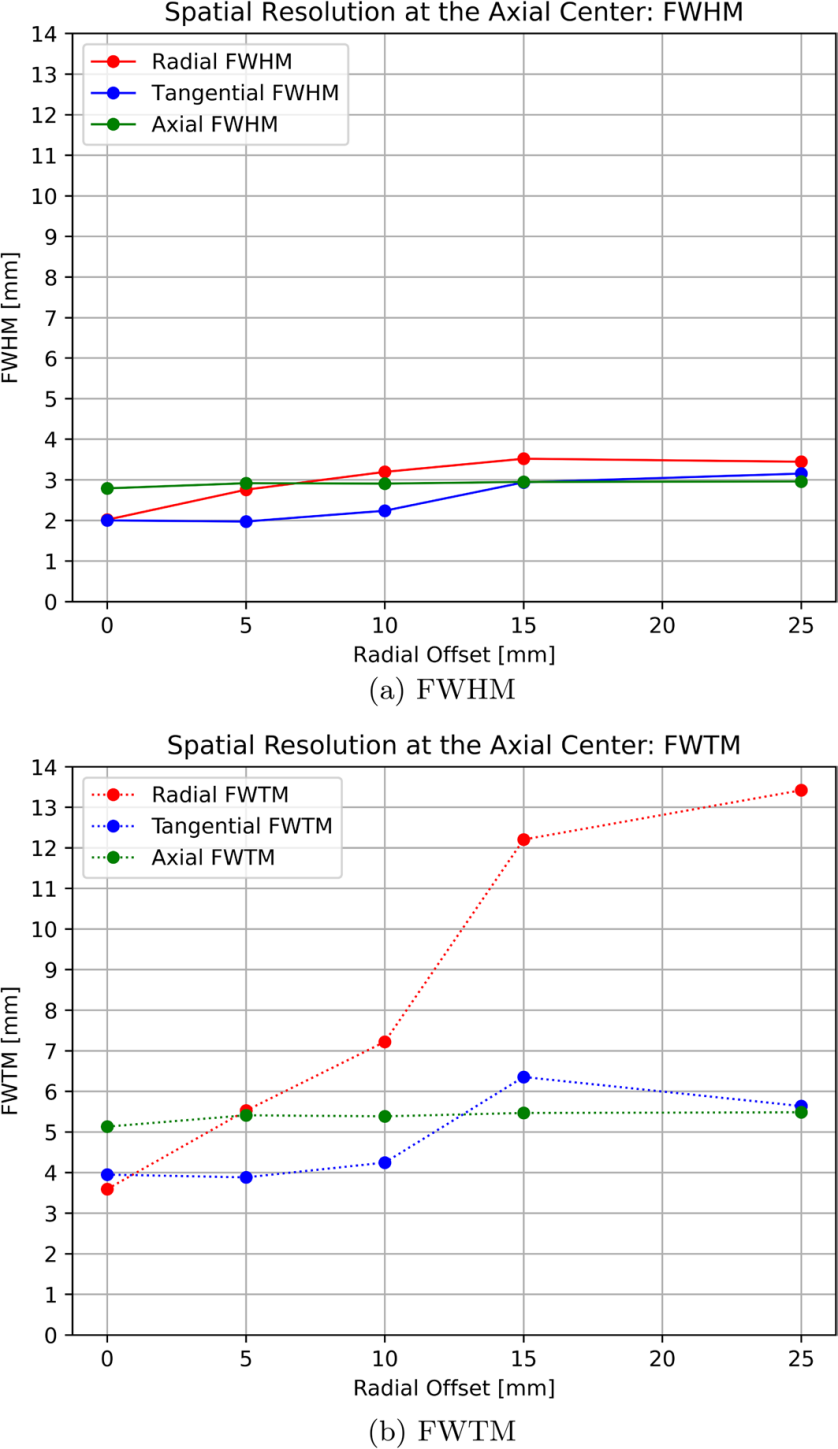
SAFIR-I spatial resolution in radial, tangential and axial direction at the center of the axial FOV with an FBP3DRP reconstruction algorithm

**Fig. 7:**
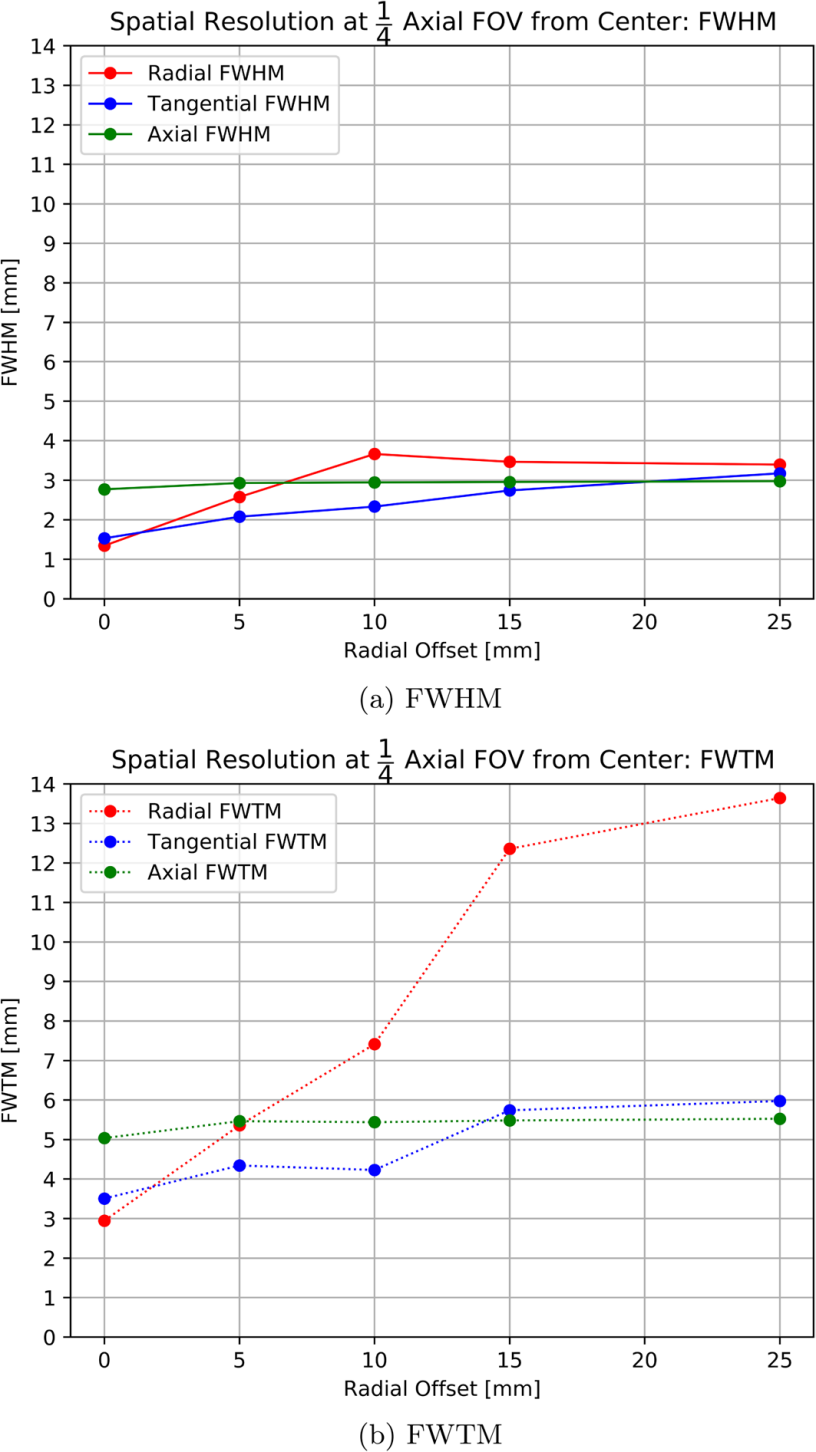
SAFIR-I spatial resolution in radial, tangential and axial direction at one quarter of the axial FOV with an FBP3DRP reconstruction algorithm

The spatial resolution results are summarized in Table 1 in terms of FWHM and Full Width at Tenth Maximum (FWTM). For better visualization, the results are also plotted in Figs. 6 and 7.

**Table 1:** SAFIR-I spatial resolution values reported in radial, tangential and axial direction in [$\hbox {mm}$] after reconstruction with an FBP3DRP algorithm

Reconstructed image pixel size [mm$^2$]: 0.55$\times$0.55										
Slice thickness [mm]: 1.1										
At axial center										
Radial offset	$0\,\hbox {mm}$		$5\,\hbox {mm}$		$10\,\hbox {mm}$		$15\,\hbox {mm}$		$25\,\hbox {mm}$	
	FWHM	FWTM	FWHM	FWTM	FWHM	FWTM	FWHM	FWTM	FWHM	FWTM
Radial	2.02	3.59	2.76	5.53	3.20	7.22	3.52	12.21	3.45	13.42
Tangential	2.00	3.92	1.97	3.88	2.24	4.25	2.94	6.36	3.15	5.64
Axial	2.79	5.13	2.92	5.41	2.91	5.39	2.95	5.47	2.96	5.49
At 1/4 axial FOV from center										
Radial	1.34	2.94	2.58	5.36	3.66	7.41	3.47	12.36	3.40	13.64
Tangential	1.53	3.51	2.08	4.34	2.33	4.23	2.74	5.74	3.17	5.98
Axial	2.77	5.03	2.93	5.47	2.94	5.44	2.96	5.48	2.98	5.53

### Scatter fraction, count losses, and random coincidence measurements results

**Fig. 8:**
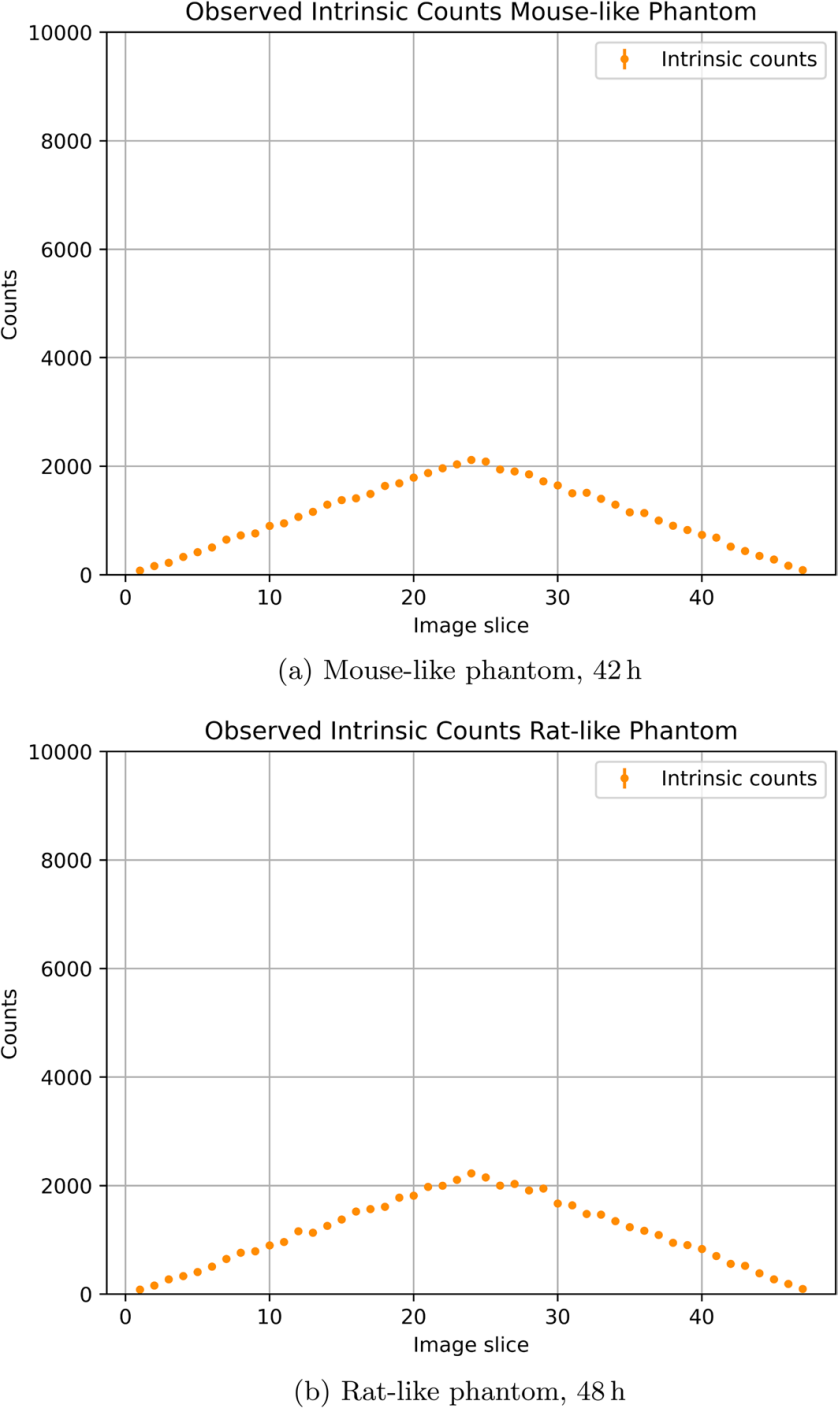
Acquired intrinsic true counts per slice, after Single Slice Rebinning (SSRB), for the mouse-like (8**a**) and rat-like (8**b**) phantoms

The absolute number of intrinsic radioactivity counts observed for the mouse- and rat-like phantoms are shown in Fig. 8. Significantly fewer than $10 000\,\hbox {counts}$ were observed in each slice over the given time period. Based on the available data, it can be estimated that an acquisition time of $221.5\,\hbox {d}$ and $241\,\hbox {d}$ would be necessary, for the mouse- and rat-like phantom, respectively, to satisfy this provision even for the outermost slices.

**Fig. 9:**
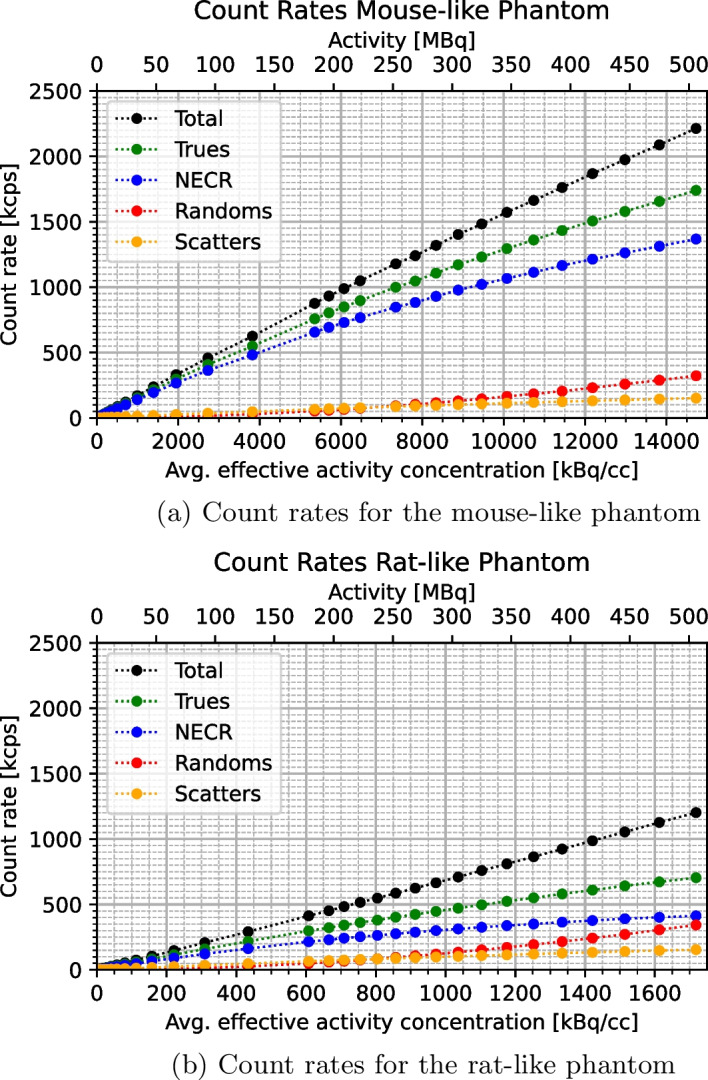
SAFIR-I count rate plots for the mouse-like and rat-like phantom

After trimming, an intrinsic true event’s counting rate of $0.03\,\hbox {cps}$ was found for both phantoms. Figure 9 then shows the count rate plots for each phantom and Table 2 lists the Scatter Fractions (SFs), together with the activities at which they are quoted.

In the case of the mouse-like phantom, the peak of neither the True Count Rate (TCR), nor the Noise-Equivalent Count Rate (NECR) was reached even at the highest activities. Accordingly, the highest observed TCR and NECR were 1740 and $1368\,\hbox {kcps}$, respectively, at the average effective activity concentration of $14.7\,\hbox {MBq}\,\hbox {cc}^{-1}$. One data point was excluded from the analysis due to a problem with the acquisition.

For the rat-like phantom the maximal values of $705\,\hbox {kcps}$ for the TCR and $413\,\hbox {kcps}$ for the NECR are found at the highest average effective activity concentration of $1.72\,\hbox {MBq}\,\hbox {cc}^{-1}$.

**Table 2:** SAFIR-I system scatter fraction report

Phantom	SF	Avg. activity
Mouse-like	0.08	$580.2\,\hbox {kBq}$
Rat-like	0.18	$564.3\,\hbox {kBq}$

### Sensitivity results

The duration required to collect 80 000 true events with the point source centered axially and transaxially, and equivalently the acquisition time for the background data set, was found to be $18.19\,\hbox {s}$.

**Fig. 10:**
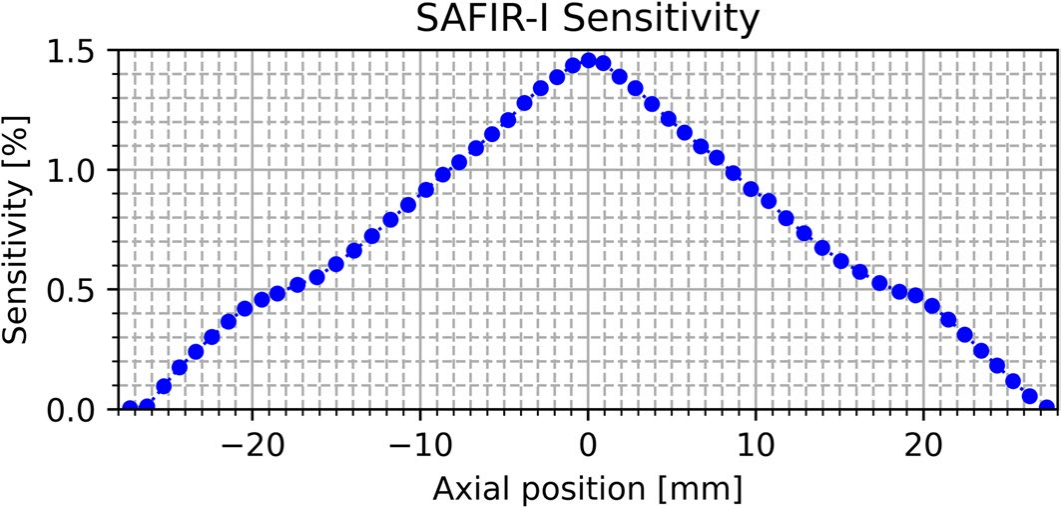
Axial sensitivity profile of SAFIR-I

The recorded sensitivities are plotted as a function of the reconstructed axial position to form the axial sensitivity profile shown in Fig. 10. For the outermost two data points on either side, i.e., the ones closest to either edge of the axial FOV, an accurate reconstruction of the axial position was not possible. To place them with reasonable accuracy, the average axial spacing between all other data points was determined ($(1.01\pm 0.07)\,\hbox {mm}$) and used to estimate the points’ axial coordinates with respect to their neighbors’. As visible in the plot, this approach works well in all positions with the exception of the outermost one in negative direction (left), which displays an apparent asymmetry; it is hence neglected for the discussion.

The peak sensitivity is $1.46\,\%$. The average system sensitivity is $0.73\,\%$. The step-number-corrected total sensitivity is $35.63\,\%$. In addition to the central peak, the profile presents two side peaks at around $\pm 18\,\hbox {mm}$.

### Image quality, accuracy of attenuation, and scatter corrections results

**Fig. 11:**
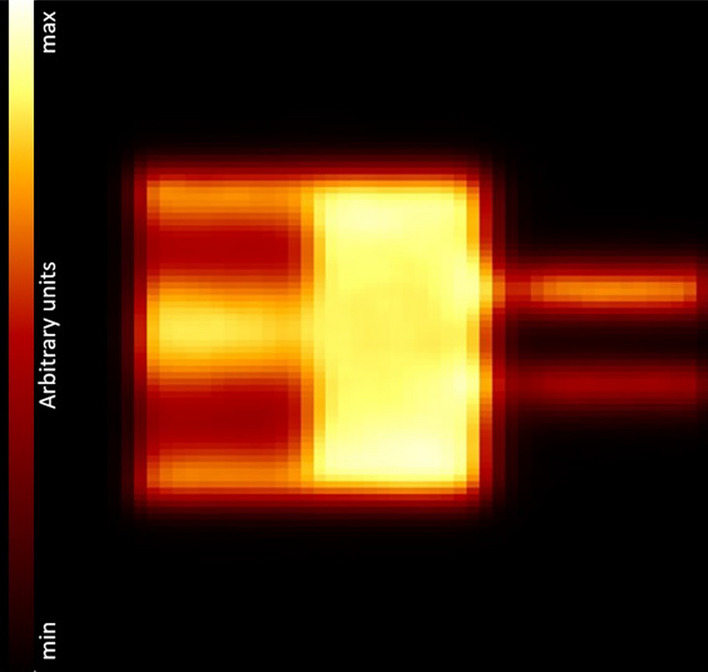
Image of the IQ phantom after MLEM reconstruction with corrections in coronal view, sliced at $4.4\,\hbox {mm}$ from the central axis in direction of the largest hot rods

**Fig. 12:**
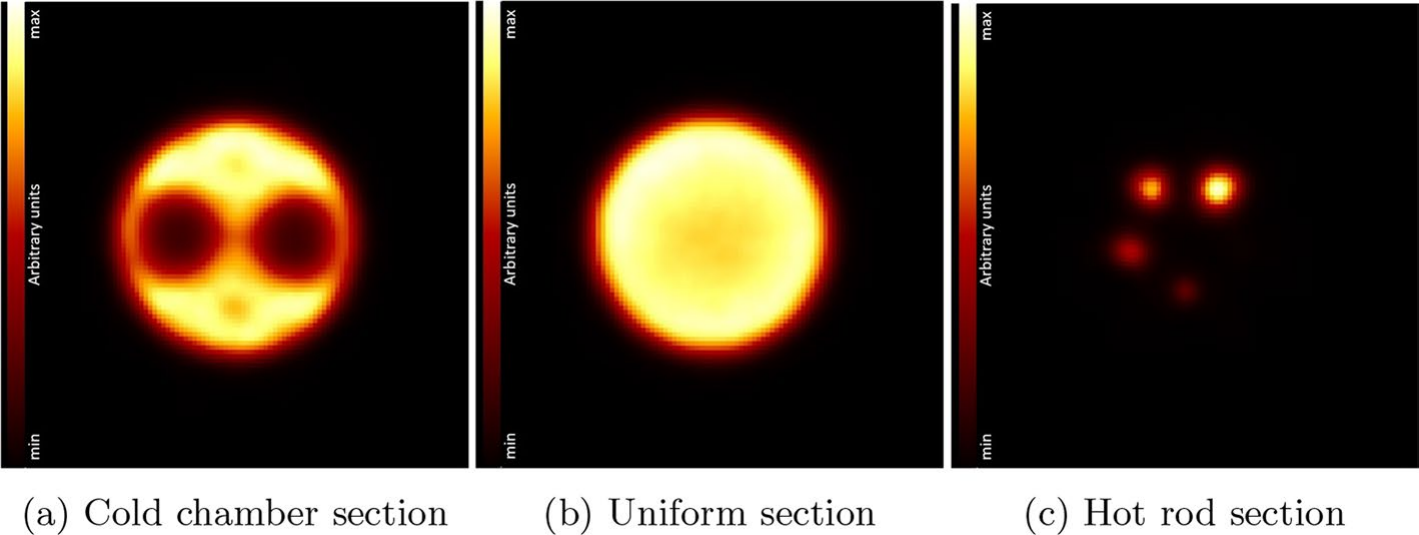
Transverse image slices through the centers of each section of the IQ phantom

A coronal cut through the reconstructed IQ phantom image is shown in Fig. 11. Additionally, three transverse cuts through the different phantom sections are shown in Fig. 12. The smallest hot rod of $1\,\hbox {mm}$ diameter could not be reconstructed (Fig. 12c). The results of the uniformity test (in calibrated counts), recovery coefficient test, and the accuracy of corrections are summarized in Tables 3, 4, and 5, respectively. The accuracy of the quantitative calibration of voxel activity was $4.3\,\%$.

**Table 3:** SAFIR-I report for uniformity test

	Mean	Maximum	Minimum	%STD
Uniformity	59064	66824	52567	4.8

**Table 4:** SAFIR-I report for Recovery Coefficient test

Rod diameter	RC	%STD
$1\,\hbox {mm}$	0.04	31.5
$2\,\hbox {mm}$	0.28	18.2
$3\,\hbox {mm}$	0.54	15.5
$4\,\hbox {mm}$	0.84	10.6
$5\,\hbox {mm}$	1.08	9.2

**Table 5:** SAFIR-I report for accuracy of (scatter) corrections

Region	SOR	%STD
Water-filled cylinder	0.220	10.9
Air-filled cylinder	0.218	9.8

## Discussion

SAFIR-I evolved from the SAFIR prototype system. The capabilities of the latter have previously been compared to a set of ten different PET systems featuring similar crystal dimensions, as well as the Bruker PET insert (Si 198) designed for use in conjunction with (e.g.,) the BioSpec 70/30 USR MRI system; the comparison showed that the prototype generally was a strong contender, however clearly outperformed in terms of achievable NECR by the MuPET (PET/Computed Tomography (CT)), Inveon (PET), Mosaic (PET; commercial name of the A-PET [18, 19]), and Bruker (PET/MRI [insert]) systems [3]. The following sections will thus repeat the performance parameter comparisons of those systems and the SAFIR prototype, in juxtaposition to the results attained with SAFIR-I.

### Spatial resolution

The spatial resolution results obtained for SAFIR-I with the FBP3DRP algorithm are consistent with the ones reported for the prototype system, both in terms of the shapes of the curves (Fig. 6), as well as in terms of absolute values reported. In both cases near-constant values in axial direction, for both FWHM and FWTM, at around $(2.9 \pm 0.1)\,\hbox {mm}$ and $5.4\,\hbox {mm}$, respectively, were found for all investigated points inside the FOV. In radial and tangential directions more gradual changes in resolution could be seen for SAFIR-I, as opposed to the small, erratic downwards fluctuations reported for the prototype system, which explains the minute difference in average resolutions for certain offsets (see Table 6). The smoother change can be attributed to the additional oblique planes resultant of the longer axial FOV. The overall upwards trend in radial FWHM is caused by the parallax error.

Compared to the selected reference scanners (see Table 6), one can see that the spatial resolutions measured for SAFIR-I are worse than the ones reported for systems using notably smaller crystals, which is consistent with the expectation of the detector size being the primary driver for spatial resolution. However, the SAFIR-I spatial resolutions are markedly better than the ones achieved by the Mosaic system, which uses crystals of comparable, yet still slightly smaller front face. The spatial resolution offered by the Bruker system, determined using MLEM reconstruction, is unmatched by any of the other systems.

Considering SAFIR-I’s irregular block detector shape, the spatial resolution can still be improved by employing alternative reconstruction algorithms like for instance MLEM, which could use the detector’s exact geometry without the need for interpolation between the blocks, hence averting the resolution-degrading streak artifacts associated with FBP3DRP in this situation [3, 12, cf.]. While the nature of a minimization algorithm can lead to an overestimate of the quality of the resolution on a point source, MLEM could give adequate estimates for extended objects in a radioactive background, e.g., the cerebral cortex inside a rat brain during an $^{18}$F-Fluorodeoxyglucose (FDG) study; in the case of SAFIR-I, this method has previously successfully been used to show that a spatial resolution of around $2\,\hbox {mm}$ can certainly be reached in the center of the FOV during concurrent PET/MR imaging [1].

**Table 6:** Average spatial resolution (FWHM in [$\hbox {mm}$]) at different radial offsets from the center of the FOV with associated reconstruction algorithms (RA) [3, 18–22]

			Radial offset			
System	RM	Crystal size [$\hbox {mm}^{3}$]	$5\,\hbox {mm}$	$10\,\hbox {mm}$	$15\,\hbox {mm}$	$25\,\hbox {mm}$
SAFIR-I	FBP3DRP	$2.12 \times 2.12 \times 13.00$	2.55	3.02	3.14	3.19
SAFIR p.s.	FBP3DRP	$2.12 \times 2.12 \times 13.00$	2.56	2.79	3.05	3.14
Mosaic	FBP3DRP	$2.0 \times 2.0 \times 10.0$	3.05$^a$	3.08$^a$	3.17$^a$	3.37$^a$
MuPET	SSRB FBP	$1.24 \times 1.40 \times 9.50$	1.27	1.30	1.36	1.43
Inveon	FORE$^b$ + 2DFBP	$1.5 \times 1.5 \times 10.0$	1.83$^a$	1.93$^a$	1.90$^a$	2.00$^a$
Bruker	MLEM	$50.0 \times 50.0 \times 10.0^{c}$	0.88	0.93	0.90	0.95

$^{a}$ Estimated from plot

$$^{b}$$ Fourier Rebinning

$$^{c}$$ monolithic crystals

### Scatter fraction, count losses, and random coincidence measurements

SAFIR-I offers excellent intrinsic suppression and count rate performance. The NECR peak is not reached even at the highest tested average effective activity concentrations, corresponding to injected activities surpassing $500\,\hbox {MBq}$, regardless of the phantom. Not considering possible systematic errors, the errors on the count rate results are estimated to be at the $3\,\%$ level, dominated by the uncertainty of the probe activity measurement in the employed calibrator. In comparison to the reference systems (see Table 7), the maximal observed SAFIR-I NECR values are now higher than the peak values reported for the Mosaic and the MuPET systems, in addition to being superior to the ones attained with the Bruker insert. Notwithstanding this NECR performance boost compared to the prototype system, SAFIR-I is still bested by the Inveon system. All four reference devices can rely on axial FOVs more than twice as long as SAFIR-I’s and reach their peak NECR values already at significantly lower activities.

The relatively short length of SAFIR-I’s axial FOV in comparison to the length of the rat-like phantom can explain the reduced number of trues found in that measurement (see Fig. 9). Assuming a perfectly uniform distribution of activity inside the phantom, roughly $61.3\,\%$ of the total activity would be outside the FOV (compared to only $9.7\,\%$ for the mouse-like phantom), thus only contributing single photons to the raw measurement data.

Based on the intrinsic coincidence rate $R_I$ and the total sensitivity $S_\mathrm{{{tot}}}$, the activity level $A_{SF}$ stipulated by the protocol for the determination of the SFs can be estimated [9, cf.]:

3 $$\begin{aligned} A_{SF}&= \Bigg (\frac{\sqrt{R_I}}{S_\mathrm{{{tot}}}} \Bigg ) \cdot 5 ~~\approx ~~ 2.4\,\hbox {Bq} \end{aligned}$$

It follows that the SFs for SAFIR-I have been established at significantly higher activities than projected by the protocol. Accordingly, recognizing a linear rise with increasing activity in both trues and scatters in the count rate plots for the low activity region, the SFs reported for SAFIR-I are likely slightly overestimated. Still, the results are comparable to the other scanners used for reference (see Table 7). In particular, they are comparable to the SFs which have been found for the Inveon system at activities $\sim 60 \times$ lower than the ones reported here [21].

**Table 7:** Peak NECR and SF comparison [3, 18–22]

	Mouse-like phantom			Rat-like phantom		
System	Max. NECR [$\hbox {kcps}$]	Activity [$\hbox {MBq}$]	SF [$\%$]	Max. NECR [$\hbox {kcps}$]	Activity [$\hbox {MBq}$]	SF [$\%$]
SAFIR-I	1368	506.1	8.0	413	506.1	18.0
SAFIR p.s.	799	537	10.9	121	624	17.8
Mosaic	308	84.5	9.6	129	100	16.8
MuPET	1100	57	11.9	352	65	28.0
Inveon	1670	131	7.8	592	110	17.2
Bruker	486	23	–$^a$	239	23	–$^a$

$$^a$$ Not reported

### Sensitivity

The sensitivity profile follows the expected triangular shape. Compared to the value reported for the SAFIR prototype, the peak sensitivity has increased by $37.5\,\%$ ($1.46\,\%$ vs. $1.06\,\%$[3]), which is comparable to the increase in solid angle coverage featured by SAFIR-I ($44.0\,\%$). The remaining difference could be resultant of the disparate source activities between the two measurements, differences between the two detectors in the configurations, as well as in the energy and timing calibrations prior to the application of respective windows, and due to the lower operating temperature (i.e., fewer dark counts) of SAFIR-I. The two side peaks correspond to the centers of the two outer rings of crystal blocks, which lie at a distance of $\pm 18.1\,\hbox {mm}$ from the axial center. This behavior is typical for multi-ring PET scanners [23, see e.g.,].

Table 8 shows a comparison to the reference scanners. The systems with significantly longer axial FOV generally achieve much higher sensitivities (e.g., the MuPET, Inveon, and Bruker systems). Considering the narrow energy window and short axial FOV, SAFIR-I has a high sensitivity, as required by its specification for high image frame rates. In particular, it can readily compete with the Mosaic system while featuring less than half the competitor’s axial coverage.

**Table 8:** Peak sensitivity comparison [3, 18–22]

System	Energy window [$\hbox {keV}$]	Time window [$\hbox {ns}$]	Axial FOV [$\hbox {mm}$]	Sensitivity [$\%$]
SAFIR-I	391 to 601	0.5	54.2	1.46
SAFIR p.s.	391 to 601	0.5	36	1.06
Mosaic	410 to 665	12	119	1.14
MuPET	350 to 650	3.4	116	6.35
Inveon	350 to 625	3.4	127	6.72
Bruker	—$^a$	—$^a$	150	11.0

$$^a$$ Not reported

### Image quality, accuracy of attenuation, and scatter corrections

The IQ phantom images are artifact-free. There is a good uniformity in the uniform hot region, with a standard deviation $\sigma$ below the $5\,\%$ level, and with the maximum and minimum values at $2.7\,\sigma$ and $2.3\,\sigma$ of the mean value, respectively. This indicates that scatter and — in particular [5]— attenuation corrections work well [9]. The uniformity is comparable to values reported for other scanners [3] including the reference scanners listed in Table 9.

The RC value of almost zero found for the $1\,\hbox {mm}$ diameter hot rod is compatible with it not being visible in the image. A full recovery ($\hbox {RC}(\%\hbox {STD})~=~1.08(9.2\,\%)$) of the introduced activity is accomplished for the $5\,\hbox {mm}$ hot rod. In general, the RCs and associated standard deviations achieved with SAFIR-I present a notable improvement over the performance reported for the prototype system [3], albeit scanners employing smaller crystals, or in the case of the Bruker insert monolithic ones, can offer even better values, especially for rod diameters smaller than $3\,\hbox {mm}$ to $4\,\hbox {mm}$ (compare Tables 6 and 9).

The SORs for the water- and air-filled cylinders are almost identical, with similar standard deviations. This can be considered another indication for the quality of the applied attenuation correction. The primary driver for this parameter can hence be considered to be scatter [9]. Further, the values are within one standard deviation compatible with the results reported for the prototype system [3], where however the observed deviations across the cylindrical volumes could be reduced by $>40\,\%$ with SAFIR-I. The reduction can be attributed to the coverage of the region with additional, more oblique planes consequent to the longer axial FOV and the resulting geometrically augmented sampling.

In direct comparison with the absolute amounts of spill-over reported for the reference systems (see again Table 9), the values determined here are undesirably higher, which could be related to the longer FOVs and better spatial resolutions offered by these systems but could also hint toward improvements still being possible for the reconstruction including corrections.

**Table 9:** Comparison of image quality parameters[3, 18–22]

			RC					SOR [$\%$]	
System	RM	%STD$_{\text {uniform}}$	$1\,\hbox {mm}$	$2\,\hbox {mm}$	$3\,\hbox {mm}$	$4\,\hbox {mm}$	$5\,\hbox {mm}$	Water-filled cyl.	Air-filled cyl.
SAFIR-I	3D MLEM	4.8	0.04	0.28	0.54	0.84	1.08	22.0	21.8
SAFIR p.s.	3D MLEM	3.0	0.13$^d$	0.29	0.49	0.65	0.88	17.3	18.5
Mosaic	3D RAMLA$^a$	5.1	0.22	0.55	0.74	0.87	0.98	6.3	2.7
MuPET	FBP3DRP$^b$	6.5	0.19	0.58$^e$	0.78$^e$	0.89$^e$	0.95	9.0	5.0
Inveon	FORE$^c$ + 2DFBP	5.3	0.17	0.48	0.72	0.84	0.93	1.7	-0.6
Bruker	3D MLEM$^b$	4.5	0.14	0.64	0.91	0.95	0.94	6.2	4.6

$^a$ Row-Action Maximum-Likelihood Algorithm

$^b$ No scatter correction applied

$$^c$$ Fourier Rebinning

$^d$ The smallest rod was not visible in the reconstructed image

$^e$ Estimated from plot

## Conclusions

The performance characteristics of the SAFIR-I PET insert have successfully been evaluated according to NEMA-NU4 under realistic conditions, i.e., with the scanner installed inside the bore of the MRI system and operated in its static $7\,\hbox {T}$ magnetic field^2^.

The spatial resolution satisfies the target specified in the SAFIR design [1, cf.] and is consistent with earlier observations on the prototype system [3].

Further, an exceptional count rate performance could be demonstrated. This result confirms SAFIR-I’s ability to handle high injected activity, which is required for the quantification of fast tracer kinetics in small animals.

SAFIR-I delivers high sensitivity, especially considering its limitations of short axial coverage, relatively large inner diameter, narrow energy window, and narrow CTW, thus facilitating rapid formation of diagnostically useful images at high activities [1, cf.] congruent with its design specification. The observed sensitivity enhancement over the SAFIR prototype matched expectations.

The IQ study proved SAFIR-I’s capabilities as a reliable quantitative imaging system. The results presented improvements over the prototype system, and demonstrated high consistency between different performance measures, as well as generally well functioning corrections. It is conceivable that the SORs could be reduced using a different scatter estimation implementation. A comparison of several reconstruction methods including STIR version 3, STIR version 5, and the internally developed Fast Tomographic Reconstruction (FTR) [4] is thus planned for the near future.

In summary, SAFIR-I delivers excellent performance, particularly at high activities, in line with its design goals. The observed capabilities allow to proceed with preclinical in vivo animal studies at injected activities reaching $500\,\hbox {MBq}$ using SAFIR-I.

Simultaneously, the efforts to improve imaging performance continue. Further investigations beyond the NEMA-NU4 protocol are required to exhaust the true performance potential of SAFIR-I in relation to chosen design parameters of an imaging study. Among many possibilities to tune the behavior of this detector are the option to include an inter-crystal scatter recovery (with a selectable number of hits detected within a user-defined radius) in the data analysis, and the option to adapt the CTW to both the activity (which influences the Coincidence Resolving Time (CRT) [1]) and the object under study. Subsequent to the comparison between reconstruction results attainable with STIR and the FTR software, an inclusion of Time-of-Flight (TOF) information is also conceivable [4, 24, cf.]. In parallel, an exploration to find the exact spatial resolution limit of the detector, e.g., by means of dedicated phantoms [25], could serve as a basis to optimize the reconstructed voxel size for reduced image noise. Finally, it could be seen in this work that the comparable shortness of the FOV introduces restrictions to the achievable performance. To overcome these limitations, the Small Animal Fast Insert for MRI detector II (SAFIR-II), featuring $144.4\,\hbox {mm}$ axial coverage, has been constructed.

## Data Availability

The data sets generated and analyzed during the current study are not publicly available due to the intellectual property rights belonging to ETH Zurich (Art. 36 Federal Act on the Federal Institutes of Technology). Data are however available from the authors upon reasonable request and with permission of ETH Zurich.

## Abbreviations

ASIC: Application Specific Integrated Circuit
CRT: Coincidence Resolving Time
CT: Computed Tomography
CTW: Coincidence Timing Window
DAQ: Data acquisition
ESR: Enhanced Specular Reflector
FBP3DRP: Filtered Backprojection, 3D Reprojection
FDG: 18F-Fluorodeoxyglucose
FOV: Field Of View
FPGA: Field-Programmable Gate Array
FTR: Fast Tomographic Reconstruction
FWHM: Full Width at Half Maximum
FWTM: Full Width at Tenth Maximum
IQ: Image quality
LYSO: Lutetium Yttrium OxyorthoSilicate
MLEM: Maximum-Likelihood Expectation-Maximization
MR: Magnetic Resonance
MRI: Magnetic Resonance Imaging
NECR: Noise-Equivalent Count Rate
NEMA: National Electrical Manufacturers Association
NEMA-NU4: NEMA Standards Publication NU 4-2008
PET: Positron Emission Tomography
PETA6SE: Position-Energy-Timing Application Specific Integrated Circuit, version 6, Single Ended
RC: Recovery Coefficient
RF: Radio Frequency
ROI: Region of interest
SAFIR: Small Animal Fast Insert for MRI
SAFIR-I: Small Animal Fast Insert for MRI detector I
SAFIR-II: Small Animal Fast Insert for MRI detector II
SF: Scatter Fraction
SiPM: Silicon Photomultiplier
SOR: Spill-Over Ratio
SP: Singles-Prompts
SSRB: Single Slice Rebinning
SSS: Single Scatter Simulation
STIR: Software for Tomographic Image Reconstruction
TCR: True Count Rate
TOF: Time-of-Flight
USR: Ultra Shield Refrigerated
VOI: Volume Of Interest

## Footnotes

1 Now Lemer Pax.

2 The radiofrequency coils were not energized. Full MR-compatibility during active runs of common MR-sequences has previously been shown [1].

## Code availability

The intellectual property rights to computer code, including firmware, developed at ETH Zurich belong to ETH Zurich (Art. 36 Federal Act on the Federal Institutes of Technology). Dedicated agreements with interested users of such material would have to be negotiated in order to make this information available.
